# Development and evaluation of a structured programme for promoting physical activity among seniors with intellectual disabilities: a study protocol for a cluster randomized trial

**DOI:** 10.1186/1471-2458-13-746

**Published:** 2013-08-12

**Authors:** Marieke van Schijndel-Speet, Heleen M Evenhuis, Pepijn van Empelen, Ruud van Wijck, Michael A Echteld

**Affiliations:** 1 Department of General Practice, Intellectual Disability Medicine, Erasmus Medical Centre Rotterdam, P.O. box 2040, Rotterdam 3000 CA, The Netherlands; 2 Ipse de Bruggen, Louis Braillelaan 42, Zoetermeer 2719 EK, The Netherlands; 3 TNO, research group Life style, Locatie leiden Gortergebouw, Wassenaarseweg 56, Leiden 2333 AL, The Netherlands; 4 Centre for Human Movement Sciences, University Medical Centre Groningen, P.O. Box 30001, Groningen 9700 RB, The Netherlands

**Keywords:** Health promotion, Intellectual disabilities, Intervention mapping, Evaluation

## Abstract

**Background:**

Older people with intellectual disabilities have very low physical activity levels. Well designed, theory-driven and evidence-based health promotion programmes for the target population are lacking. This paper describes the design of a cluster-randomised trial for a systematically developed health promotion programme aimed at improving physical activity and increasing fitness among seniors with intellectual disabilities.

**Methods and design:**

The Intervention Mapping protocol was used for programme development. After defining the programme’s objectives, the following behavioural techniques were selected to achieve them: Tailoring, Education, Modelling, Mirroring, Feedback, Reinforcement and Grading. With professionals and managers of provider services for people with intellectual disabilities, we translated these strategies into a structured day-activity programme, that consisted of a physical activity and an education programme. The programme will be executed in five day-activity centres in groups of eight to ten seniors during eight months, whereas seniors in five other centres receive care as usual. The physical activity level, as measured in number of steps a day, will be used as primary outcome measurement. Secondary outcome measurements include motor fitness, cardio respiratory fitness, morphological and metabolic fitness, ADL, functional deterioration and depressive symptoms. Differences in the primary and secondary outcome measures between participants and controls will be analysed using generalized estimation equations, correcting for day-activity center as cluster.

**Discussion:**

This paper provides insight into the development and content of a theory-driven intervention aimed at behavioural change in a population with a low intellectual level. Its evaluation design is described. The programme’s applicability to other populations is discussed.

**Trial registration:**

Trial number: ISRCTN82341588

## Background

It is a well-known fact that people with a low education level and socioeconomic status generally tend to have an unhealthy lifestyle, whereas community-based programmes aimed at improvement of healthy behaviour are insufficiently effective in reaching this population group [1]. This is even more so in adults with intellectual disabilities (ID), i.e. with IQ levels below 70 before the age of 18 and limitations in adaptive behavior. In Dutch adults with ID aged 50 years and over, a large majority has a sedentary lifestyle and fitness levels comparable to the general population aged 80 years and over [2], leading to increased prevalences of obesity, diabetes, metabolic syndrome and other age-related health risks [3]. Although in practice, several physical activity programmes have been developed for Dutch adults with ID, none of them have explicitly been designed using behavioural change theory or have been scientifically evaluated. To our knowledge, internationally, only one such programme has been set up for young adults with a mild ID and evaluated properly [4].

An approach based on theories and strategies for behavioural change is considered the initial step in the development and evaluation of complex interventions, increasing the likelihood of the interventions being effective [5,6]. Although several theory- and evidence-based programmes to increase physical activity are available for older adults in the general population [7,8], the applicability of such programmes to older people with ID is limited, because of their limited cognitive ability, frequent mobility and sensory limitations or other health problems, need of support, and limited financial means [9-11].

In the current report we describe the design of the evaluation study for a theory-based programme to promote physical activity and fitness in seniors with ID. Because persons with severe and profound ID (IQ lower than 35) cannot be verbally instructed and require a completely different approach, we decided to start with a programme for people with mild (IQ 55–70) and moderate (IQ 35–55) intellectual disabilities.

## Methods and design

This study will evaluate the efficacy of the “Healthy Ageing- Physical Activity Programme (HA-PAP) for seniors with Intellectual Disabilities”. More specifically, physical activity, fitness and health indices will be compared between older adults with mild-moderate intellectual disabilities (ID) who are assigned to the HA-PAP and who will be given usual care. The programme is developed to be executed in day-activity centres of care organisations for people with ID in the Netherlands, in groups of eight to ten seniors. Intervention Mapping (IM) was applied to systematically develop the programme [12]. The efficacy of the programme will be evaluated in a cluster-randomized clinical trial and to minimize contamination, the level of day centres is used as the randomization level.

HA-PAP was approved by the Medical Ethics Committee of the Erasmus University Centre Rotterdam (NL 29573.078.09).

### Development of the programme

We applied Intervention Mapping (IM) to systematically develop the programme [12]. Intervention Mapping is a six-step protocol that facilitates a procedure for theory-based and evidence-based development of heath promotion interventions. In the first two steps, an assessment of needs and programme objectives, including a specification of necessary behavioural changes, are described. In step three, theory-based strategies are selected, which form the basis of the intervention programme, developed in step four. In this phase, we also selected and/or produced intervention materials. We will describe how the subsequent steps were addressed in the current study, resulting in our choices of goals, theories and strategies, applicable to this specific low IQ population.

Step 1: Needs assessment

Information on factors that may directly or indirectly influence participation in physical activity of this population was collected from the literature, by consultation of managers and movement experts of ID care provider services, and by 14 in-depth interviews and four focus-group discussions with seniors with mild and moderate ID. The interviews with the seniors themselves provided information regarding their preferences for specific physical activities and revealed 30 factors that promote or hamper participation in physical activity, the details of which will be published elsewhere (Van Schijndel-Speet M, Evenhuis HM, Van Wijck R, Van Empelen P, Echteld MA: Facilitators and barriers to physical activity as perceived by older adults with intellectual disabilities, submitted). The information obtained through these activities formed the basis for all further steps of the IM protocol.

When looking at the barriers for older adults with ID regarding physical activity, two important overarching barriers became apparent from the literature and were confirmed by managers and movement experts: a lack of social support [13-15] and the need of specific activities [16-19]. A third important barrier, mentioned by managers and movement experts, was the need for professional support. Enjoyment in physical activity in addition, was a recurrent theme in the interviews with seniors with ID themselves. The facilitators they mentioned were also found in studies among younger adults with ID [13-15] and confirmed by movement experts of the ID care provider services. These needs for social support, specific activities, professional support and enjoyment in activities will be elucidated in this paragraph.

Social support is a robust stimulus for physical activity for everyone, but specifically for seniors with ID. They need to be supported in activities themselves, or in the transfer to the activities, and they mostly do not think of the possibility or have the desire to become physically active themselves [13,14]. Staff members in the residential setting however do not always have enough time or are insufficiently aware of the importance of regular physical activity for their clients [14]. Support by family may be limited after parents have died [16]. Aiming at increasing physical activity among the target group therefore means that we have to make sure that staff is motivated and sufficient staff is structurally available.

Managers and movement experts of the ID care provider services underlined that older adults with ID are in need of specific activities adapted to their age and physical limitations. As a result of childhood disabilities and multi morbidity, decline in physical functioning may start at a younger age when compared to seniors with normal intelligence [18]. Physical limitations such as balance and coordination problems, decrease in mobility and muscle strength [20], combined with limited understanding and often limited experiences with physical activities [15] require professional support to create a safe environment and to select appropriate activities for the group concerned. Such professional support can be provided by physical therapists or physical activity instructors, experienced in conducting physical activities with (older) adults with ID.

Seniors prefer activities they enjoy and feel comfortable with. Physical activities therefore should connect with their interests, should be part of their daily routine, should be well feasible for them (so they can become good at it and get rewarded), should include coffee breaks and should be conducted together with peers in a pleasant atmosphere. Furthermore, seniors with ID prefer to participate in activities close to their homes to avoid transportation problems and activities should be affordable.

In conclusion, seniors with ID have low physical activity and fitness levels and need to become more active for the benefit of their health and well being. There are specific needs that have to be fulfilled to develop an effective and successful physical activity programme as described in this paragraph. These needs were taken into account in steps three and four addressing the development of the programme.

Step 2: Programme objectives

In the second step of IM, we distinguished an overall health goal, performance objectives and change objectives.

#### Overall health goal

In accordance with the general health goal [21], our programme’s goal was to increase the physical activity level of seniors with a mild or moderate ID who walk less than 7500 steps a day, and to maintain or increase the physical activity level of those who walk more than 7500 steps a day. In addition, the increased physical activity should lead to a delay of the decline of physical fitness, or even increase the physical fitness level.

#### Performance objectives

Performance objectives are objectives that need to be addressed in order to achieve the overall programme goal. Based on theories suggesting that behavioural change can be differentiated into a pre-actional/intentional stage, an actional stage, and a maintenance stage [22,23], we defined three specific performance objectives at an individual level: 1) Seniors *decide to* participate in physical activities offered at the day-activity centre; 2) Seniors *participate actively* in physical activities offered at the day-activity centre; 3) Seniors *maintain participating* in physical activities at the day-activity centre and maintain other physical activities they were used to do.

#### Change objectives

Change objectives are changeable preconditions that have to be fulfilled in order to ensure active participation in a behavioural change programme. Information on personal and psychological barriers that had to be overcome and facilitators that could be used to ensure that seniors would participate actively were derived from the in-depth interviews and focus-group interviews with the target group (Van Schijndel-Speet M, Evenhuis HM, Van Wijck R, Van Empelen P, Echteld MA: Facilitators and barriers to physical activity as perceived by older adults with intellectual disabilities, submitted). According to the Theory of Planned Behaviour [24], we structured these factors according to attitude, self-confidence and social support (second column of Table 1). We subsequently translated each factor in change objectives that have to be achieved to ensure active participation in physical activities, such as feeling physically safe and comfortable to perform physical activities (shown in the third column of Table 1).

Step 3: Theory-driven strategies

**Table 1:** Objectives and determinants for increasing or maintaining the participants’ physical activity (PA) level during the three phases of the study

**Performance objectives**	**Determinants**		**Change objectives**
Phase 1 Seniors decide to participate in physical activities offered at the day -activity centre	Attitude	+ enjoyment of activity	a) seniors think it is fun to participate in PA
	Self –confidence	- lack of self-confidence (lack of skills, fear of falling)	b) seniors think they are able to perform PA
		- physical complaints/pain	c) seniors think it is physically safe and comfortable to perform PA
		- feeling tired	
	Social support	- lack of social support	d) seniors feel stimulated and supported by others to perform PA
		- feeling insecure social context	
		+ activity with familiar others	
		+ pleasant atmosphere	
Phase 2 Seniors participate actively in physical activities offered at the day- activity centre and maintain participating in activities they were used to doing	Attitude	+ enjoyment of activity	a) seniors enjoy participating in PA
		+ familiarity/routine	b) seniors experience PA as part of their daily activities/routine
		+ aware of advantages of PA to their body	c) seniors become more consciousness of benefits of being PA
			d) seniors learn about normal bodily reactions to PA
	Self – confidence	+ self-confidence	e) seniors experience they are able to perform PA
		- physical complaints/pain	f) seniors feel physically safe and comfortable to perform PA and explore their skills
		- feeling tired	
	Social support	+ social support	g) seniors feel stimulated and supported by others to perform PA
		+ status of physical activity	h) seniors are proud of their achievement
		+ activity with familiar others	
Phase 3 Seniors maintain participating in physical activities at the day-activity centre and other activities they were used to doing	Attitude	+ enjoyment of activity	a)seniors enjoy participating in PA
		+ familiarity/routine	b) seniors experience PA as part of their daily activities/routine
	Self –confidence	+ self-confidence	c) seniors know they are able to perform PA
	Social support	+ social support	d) seniors feel stimulated and supported by others to perform PA

In the third step of IM, theoretical models and practical strategies that have been identified in previous research to be likely to change the identified determinants, were selected for the design and execution of the programme. The selection was again based on behavioural change theory, such as Social Cognitive Theory [25] and the Theory of Planned Behaviour [24]. The following theoretical methods were selected: tailoring, education, grading, modelling, feedback, mirroring and reinforcement. Definitions and theories have been derived from an overview of behavioural change techniques used in interventions [26].

*Tailoring*[27]*:* adapting communication and activities to one specific person

*Provide information on consequences of behaviour in general (Theory of Planned Behaviour)*: providing information about health benefits of physical activity and immediate (negative) bodily reactions to physical activity.

*Set graded tasks* (*Social Cognitive Theory*): breaking down the target behaviour into smaller and easier to achieve tasks.

*Provide instruction and model/demonstrate behaviour* (*Social Cognitive Theory)*: a leader serves as an example to the client, provides instructions about the desired behaviour and shows how to correctly perform the behaviour.

*Provide feedback on performance: (Control Theory)*: provide information about the behavioural performance of the person.

*Mirroring (Social Cognitive Theory):* copying the desired behaviour of the programme leader and peers.

*Prompt rewards contingent on effort or progress towards behaviour and on successful behaviour (Operant Conditioning)*: praise, encouragement or material rewards that are explicitly linked to the achievement of the specific behaviours or for *attempts* at achieving a behavioural goal.

To ensure achievement of the overall health goal, we further studied evidence-based guidelines for physical activities designed to achieve health benefits. Because no specific guidelines exist for physical activity for people with ID, we adopted the guidelines of The American College of Sports Medicine (ACSM) and the American Heart Association (AHA) [28,29] for the chronically ill and people aged over 65 years for our programme. Multi-component exercise programmes, including aerobic endurance, strength, balance and flexibility are recommended and optimal frequency, intensity and duration of the activities are specified.

Step 4: Development of programme and materials

Based on the identified needs of and objectives for seniors with ID, we decided to develop a structured physical activity programme to be integrated into the day-activity programme. In contrast to the home setting, staff in day-activity centres would be structurally available to support seniors participating in physical activities. Implementing the programme into the day-activity setting would provide structural physical activity integrated in their daily routine, in a familiar environment and transportation problems could also be mitigated.

We translated the selected behavioural change techniques and the physical activity guidelines into practical strategies (see Table 2, third column). With regard to the strategy ‘to provide information about performing physical activity safely and about normal reactions’ we decided with members of the project group (see step 5) to develop an education programme and a physical activity programme. Managers of day-activity centres and movement experts of ID-care provider services recommended an optimal group size of eight to ten seniors. To ensure the feasibility of the programme, we derived relevant information from the interviews with the target population, e.g. about preferences for physical activities, and developed the intervention in collaboration with professionals from the ID provider services.

**Table 2:** Behaviour change techniques and strategies to promote physical activity (PA) used in the three phases of the study

***Change objectives***	***Behaviour change techniques***	***Strategies***	***Phase 1***	***Phase 2***	***Phase 3***
Seniors enjoy participating in PA	Tailoring	Professionals select PA on preference target group	*X*	*X*	*X*
Seniors think it is physically safe and comfortable to perform PA activities	Provide information on consequences of behaviour in general	Day-activity centres’ staff members apply several work forms and exercises in groups to provide information about performing PA safely and about normal bodily reactions to PA	*X*	*X*	
	Professional support	Support PA by *familiar* professionals	*X*	*X*	
Seniors experience PA as part of their daily activities/routine	Repetition	Structured PA in the day-activity programme, three times a week		*X*	*X*
Seniors think they are able to perform PA	Set graded tasks	Select relatively easy, low intensive, short duration PA in Phase 1. Incremental increases follow.	*X*	*X*	
	Provide instruction on how to perform behaviour	Professionals tell seniors how to perform the PA	*X*	*X*	*X*
	Model/Demonstrate behaviour/	Professionals show seniors how to perform the PA through demonstrations.	*X*	*X*	
	Mirroring	The PA takes place in a group setting, where other participants demonstrate PA.	*X*	*X*	
Seniors become more conscious of the benefits of being physically active	Provide information on consequences of behaviour in general	Day-activity centres’ staff members apply several work forms and exercises in groups to provide information about benefits of PA		*X*	
Seniors learn about normal bodily reactions to PA	Provide information on consequences of behaviour in general	Day-activity centres’ staff members apply several work forms and exercises in groups to provide information about normal bodily reactions to PA	*X*	*X*	
Seniors feel physically safe and comfortable to explore their skills	Professional support	Support PA by familiar professionals	*X*	*X*	*X*
Seniors feel stimulated and supported by others to perform physical activity	Prompt rewards contingent on effort or progress towards behaviour	Using praise and rewards for attempts at performing PA.	*X*	*X*	
	Provide rewards contingent on successful behaviour	Professionals and day-activity centres’ staff members reinforce successful performance of performance of PA. This includes praise, encouragement and material rewards.	*X*	*X*	*X*
	Provide feedback on performance	Providing information about participants’ progress in performing the PA.	*X*	*X*	
	Plan social support/change	The PA take place in a group setting, with peers familiar to the participant, in which social support can be encouraged.	*X*	*X*	*X*

#### Education programme

The education programme was inspired by the health promotion programme: “Health Matters”, developed for adults with mild ID [30]. Like our education programme, the education curriculum of Marks et al. (2010) focuses on the increasing consciousness of normal bodily reactions to physical activity, the importance of physical activity for health and making healthy lifestyle choices. Because the content of the exercises was too difficult for our target group, we focused on the first two goals and developed exercises that relied on ‘experiencing’ the activity. For example, within the theme ‘breathing’ the participants will be asked to “blow a feather over the table” and in another activity we will ask them “to lay down on the floor and see your tummy going up and down”. Information derived from the interviews was used for adaptations, e.g. resulting in a theme: ‘Fear of falling’ (Van Schijndel-Speet M, Evenhuis HM, Van Wijck R, Van Empelen P, Echteld MA: Facilitators and barriers to physical activity as perceived by older adults with intellectual disabilities, submitted). Our programme was developed with experts in educating people with ID and developing suitable educational materials. The final programme includes 13 themes, for example: “What is physical activity”, “My body, the inside”, “My body, the outside”, “Fear of falling” and “My heart rate”. Specific goals for each theme were described and four to eight activities per theme were developed to achieve the goals. Selected didactical methods are sorting/ranking, group discussion, games, creative activities and role-play.

The day-activity centres’ staff members, the programme’s executors, will choose activities and didactical methods that correspond to the groups’ level of functioning. If necessary, they can adapt exercises to individuals’ level of functioning. Discussion of the participants’ experiences with the physical activities is part of the education programme. Materials such as pictograms and pictures are selected or specifically developed to support the communication about the themes and the explication of the activities. Written information about the theme, including pictures, pictograms and exercise worksheets, can be included in the participants’ individual portfolios.

#### Physical activity programme

Together with movement experts, physical activity-instructors and physical therapists who work with the population concerned, a physical activity framework was developed in accordance with the ACSM and AHA guideline. Although the guideline specifies exercise frequencies of at least five days a week, we chose a programme frequency of three times a week, taking the burden for the seniors and staff into account. In consultation with the movement experts, we selected feasible activities for each of the four fitness components: 14 exercises for endurance, 18 for strength, 17 for balance and 6 for flexibility. Each selected activity was structured, i.e.: goal, duration, required setting, materials, conduction and suggestions for safety.

Information about the feasibility of the ACSM and AHA guideline among seniors with ID is lacking, specifically concerning the duration and intensity of the physical activities. Therefore, we decided to start with simple activities and subsequently grade the activities step by step in duration and intensity (Table 3). We will start with a total programme duration of 15–20 minutes and subsequently expand the duration to 45 minutes. During each exercise period, intensity will be monitored by a heart rate monitor so that we are able to decide what intensity levels are feasible for each individual participant. If physical activity-instructors observe that seniors are not motivated to participate (more) actively, they can try to identify potential barriers that the seniors are experiencing and subsequently try to enlarge their self-confidence and enjoyment in participating, if necessary at a lower intensity.

**Table 3:** Framework of the physical activity programme

	**Day 1**		**Day 2**		**Day 3**	
	*Endurance*		*Endurance*		*Endurance*	
**Initiation (6 wks)**	10 min	HRR^*^ 20-30%	15 min	HRR 20-30%	10 min	HRR 20-30%
**Action/Extension (14 wks)**	15-20 min	HRR 40-60%	20-30 min	HRR 40-60%	15-20 min	HRR 40-60%
**Maintenance (12 wks)**	15-20 min	HRR 40-60%	20-30 min	HRR 40-60%	15-20 min	HRR 40-60%
	*Strength*		*Balance*		*Strength*	*Balance*
**Initiation (6 wks)**	10 min		5 min		10 min	5 min
**Action/Extension (14 wks)**	15 min		10 min		15 min	10 min
**Maintenance (12 wks)**	15 min		10 min		15 min	10 min
	*Flexibility*		*Flexibility*		*Flexibility*	
**All phases**	5 min		5 min		5 min	

^*^Heart rate reserve (HRR) the difference between a person’s measured or predicted maximum heart rate and resting heart rate.

Physical activity-instructors are responsible for the contents of the activity programme and its adjustment to the specific interests and needs of a group. They are also responsible for executing the programme correctly and safely, as well as for the application of the behavioural strategies selected in Step Three. Day-activity centres’ staff members will be coached by the physical activity-instructors in conducting the physical activities. They also will serve as role models and supported and encourage the participants. Positive feedback and rewards such as medals and stickers will be provided to increase the participants’ enjoyment in the activities and to motivate them. Because the physical activities are group-orientated, observing and learning from peers performing the physical activities is implicit part of the activities.

### Implementation and evaluation of the programme

In the last two steps of the Intervention Mapping protocol, implementation and evaluation plans are developed.

Step 5: Implementation.

#### Selection of day-activity centres and participants

This study is conducted by a Dutch consortium of three large ID care provider services and the Intellectual Disability Medicine research group of the Erasmus Medical Centre at Rotterdam, in collaboration with the Centre of Human Movement Sciences of the University Medical Centre Groningen. HA-PAP is set in the three large ID care provider services, that include nineteen day-activity centres offering recreational activities to the target group. Two day-activity centres with reorganization problems or substantial personnel problems are excluded. Based on earlier research, we expect to obtain informed consent for about 50% of the participants [31]. Because we planned to conduct the intervention in groups of eight to ten seniors (see Paragraph: development of the programme), 7 day-activity centres with less than 15 seniors with mild or moderate ID are excluded. The ten remaining centres all have a large activity-room or a gymnasium facility in-house or nearby.

Our primary outcome measure is physical activity, defined as steps per day. We have data of seniors with a mild or moderate ID from the pilot of the study: “Healthy Ageing with Intellectual Disabilities” (HA-ID) [31] at our disposal (n = 37), in which we found a mean physical activity level of 5480 steps per day (SD 2146). With a power of 80% to detect a difference of 1073 steps (effect size 0.5) and a type I error of 0.10, 60 seniors in both the control group and the participation group are required. We assume a drop out of 20% and planned to start therefore with 80 seniors in each group.

Seniors had to be aged 45 years and over and to be able to participate in groups. Clients who were dependent on a wheelchair inside, who had dementia or had a medical contra-indication for participating in the physical activity programme would be excluded from the study. Managers of the selected ten day-activity centers provided anonymous information about their clients on date of birth, gender, level of intellectual disability, use of walking aid, presence of dementia and the ability to participate in group activities. Clients who satisfied the inclusion criteria are selected (n = 237). Clients who are competent according to their behavioral therapist, will be informed about the project by the staff members who are familiar to them. The information will be structured using specific Patient Information Forms and subsequently written informed consent will be obtained. If older adults are not sufficiently competent to give informed consent themselves, Patient Information Forms and informed consent forms will be sent by mail to their legal representatives. Informed consent for blood sampling will be obtained separately and is no precondition for participation.

#### Randomization

To help ensure comparability of the participants conducting the programme and the seniors in the control group, we looked for the best possible match with respect to sex, age, intellectual disability, the use of a walking aid and provider service. The matching procedure was conducted with the data of the potential 237 participants (see previous Paragraph) using chi-square and t-tests. The best match was found when the ten day-activity centers were divided into two specific groups of five centres each. We subsequently checked the comparability of important day-activity centres’ characteristics in both groups, that is the availability of a gymnasium nearby and involvement of a physical-activity instructor in the day-activity centres’ activities. These characteristics appeared to be comparable. The chairman of the steering group, in presence of the researcher and a senior staff member Public Relations, randomly selected one of the two groups of five day-activity centers who will conduct the programme. The remaining group of five day-activity centres will serve as the control group. Because the implementation of the programme has to be prepared and organized, the sequence is only concealed for the clients and family, not to the day-activity centres’ managers. After the informed consent procedure has ended, all participants and/or their family will receive a confirmation of participation in the study and information about the intervention allocation.

#### Forums for implementation

Several forums are formed for the purpose of optimal motivation and support. Managers of the three ID care provider services regularly met with the research group to discuss the preconditions for implementation of the programme. Client boards were informed at the beginning of the study. A central *project group* is formed at the beginning of the study, to advise the researchers during every step of implementation of the programme. The project group consists of day-activity centres’ managers and day-activity centre’s staff, movement experts and a behavioural therapist. During the programme’s development phase, we already examined the potential fit with the participants and with the setting in which the programme needed to be carried out. A *linkage board i*s formed, responsible for the execution of the implementation plan. This linkage board consists of the researcher, a care director and a middle manager of the participating ID care provider services. Staff members in the homes of potential participants will be informed about the study and the importance of physical activity for their clients by their manager and researchers.

#### Materials and training

The programme will be implemented in five day-activity centres, the other five day-activity centres participate as control group. Together with the movement experts, a list of basic low-cost materials to perform the physical activities is drafted and purchased, including heart rate monitors. We developed a one-day training for physical activity-instructors, with the aim to teach them about the physical activity framework and how to design the physical activity programmes, that address the needs and interests of the target group, and is consistent with the underlying programme theories. They will also learn how to apply the heart rate monitors. The staff of the participating day-activity centres also will receive a one-day training, developed by a professional trainer, about the education programme. They receive information about why physical activity is important for their clients and about the goals and structure of the education programme. In addition, they will practice preparing and conducting the exercises and how to adapt exercises to the needs of their group.

#### Monitoring execution of the programme

A *workgroup* will be formed in each day-activity centre that consists of the programme’s conductors, a physical therapist, a behavioural therapist and the researcher. This working party will meet every five to six weeks to evaluate the execution of the programme and establish solutions for the barriers that will be presented. Four months after the intervention has started, two evaluation meetings will be held to discuss barriers they met and exchange successful strategies for optimal participation of the seniors. One concerning the education programme with all staff members of the five participating day-activity centres and one with all the movement experts, involved with executing the physical activity programme.

Step 6: Evaluation

The evaluation includes an effect evaluation and a process evaluation. The main outcome variable in the effect evaluation is physical activity level, and will be measured with a pedometer that will be worn for at least 4 days [32]. The NL-1000 (New-Lifestyles, Missouri USA) is a hip-worn device that measures vertical acceleration, using a piezo-electric accelerometer mechanism. It measures reliably at a walking speed of 3.2 km/h or higher [33,34]. Secondary outcome measurements include: muscle strength, balance, walking speed, blood pressure, aerobic capacity, weight, waist circumference, serum glucose, serum cholesterol, mobility, daily living skills, depressive symptoms and functional deterioration. Measurement instruments that are selected in the epidemiological study HA-ID and that were proven to be reliable among people with ID [31] are used for data collection.

All outcomes will be measured at baseline and after eight months, at the end of the intervention period. Health outcomes (motor fitness, cardio respiratory fitness and morphological fitness) will be assessed in or near the day-activity centers and performed by specially trained physical therapists and medical assistants, experienced in supporting seniors with ID [31]. Caregivers working at the homes of the participants and behavioral therapists will receive several checklists and/or questionnaires by mail (about mobility, daily living skills, depressive symptoms and functional deterioration).

The process evaluation will entail analysis of data on the attendance, barriers, initial use, exposure and continued use after the intervention period. Physical activity-instructors will fill in registration forms during the intervention period about e.g. attendance and participation of each senior and the content and duration of each physical activity. Participants, programme leaders, and managers will be interviewed and/or receive a written questionnaire about barriers and continued use of the programme at the end of the intervention period.

#### Analysis

Generalized estimation equations will be used to identify significant differences in the primary and secondary outcome measures between participants and controls, correcting for day-activity centre as cluster. The outcomes at the end of the intervention for the control group and participation group will be compared, adjusted for outcome differences at baseline.

## Discussion

The development of a day-activity programme to increase physical activity among seniors with mild or moderate ID by following the six steps of Intervention Mapping has resulted in a novel, theory-driven intervention based on strategies proven to be effective in other populations. The programme will be evaluated using a cluster-randomized trial. Analyses will control for clusters and differences at baseline. The programme’s five most important characteristics are 1) Structured multi-component physical activity programme based on the evidence-based guidelines, 2) Embedded in a routine day-activity programme, 3) Executed in peer groups, 4) Conducted by specifically trained movement experts and staff, 5) Supported by an education programme. Next to education, several behavioural change techniques are used to achieve the desired results, including: “Set graded tasks”, “Provide instruction and model/demonstrate behaviour”, Provide feedback on performance”, “Mirroring” and “Prompt rewards contingent on effort or progress towards behaviour and on successful behaviour”. The design of the programme is such that its contents can be tailored to the individual participant. An effect and process evaluation is being performed in a randomized clinical trial design.

Seniors with ID, experts in working with seniors with ID and managers involved with the implementation of the intervention, have had an important influence on the main concepts of the underlying programme. The experts who will conduct the programme played a central role in putting together a suitable programme and will offer the exercises using methods and strategies that fit best to the capacities, skills and needs of the participants with ID. By organising the intervention in the day-activity setting, there are no extra costs for the participants, the activities are nearby, and staff is available to support them in performing the activities. Although more health or fitness effects might be attained by individual fitness programmes with specific equipment, such a high-cost programme has no chance of being implemented on the long run. By selecting group activities and low-cost materials, we increased the feasibility and the likelihood of the programme’s future implementation. Also the fact that the developed programme allows the programme leaders to tailor the programme to the abilities and interests of the target group, permits a broad implementation.

We found that Intervention Mapping is a useful tool to develop a theory-based programme, including strategies that appeared effective in other subgroups. To our knowledge, the used physical activity guidelines and behavioural change techniques have never been applied to and evaluated in older persons with ID. Therefore, the programme’s effectiveness still needs to be evaluated. Although health promotion research and practice projects have been expanding over the last few years in the field of people with ID, little research has focused on the development and evaluation of theory-based interventions and explicit behavioural change techniques [35,36]. Using but also reporting the underlying theory and behavior change techniques are valuable, because it contributes to the possibility of replicating effective interventions, synthesizing evidence and understanding causal mechanisms underlying behaviour change [26,37]. Marks et al. (2010) developed a health promotion programme based on Social Cognitive Theory and the Transtheoretical model, targeted to adults with mild intellectual disabilities living in the community with limited support. The activity programme included activities in the gym, which were not applicable to our study group, because of lack of fitness equipment in day-activity centres and because these activities could not be conducted in groups of eight to ten older adults with ID.

In conclusion, the HA-PAP programme, is a day-activity programme consisting of a physical activity programme and an education programme. The effect evaluation of the study will provide insight into the programme’s effects on physical activity and health. The process evaluation will provide more insight into the attitudes, facilitators and barriers to the implementation of the programme. If the intervention proves to be effective, a well-developed theory and evidence-based day-activity programme will become available for the promotion of physical activity among seniors with ID. Although this programme was developed explicitly for seniors with ID, we believe that the main programme elements may also be suitable for other subpopulations with similar characteristics, such as older people living in a residential setting and/or people with low-education levels.

## Abbreviations

ACSM: American college of sports medicine; AHA: American heart association; HRR: Heart rate reserve; ID: Intellectual disabilities; IM: Intervention mapping; HA-ID: Healthy ageing with intellectual disabilities; HA-PAP: Healthy ageing- physical activity programme for seniors with intellectual disabilities; PA: Physical activity.

## Competing interests

The authors declare they have no competing interests.

This study was carried out with the financial support of the ZonMw (grant no.57000003), with no involvement in recruitment, data collection, analyses or interpretation of data, in writing the article or in submission for publication.

## Authors’ contributions

MvS collected the data and drafted the manuscript. HE is supervisor of the study and revised the manuscript critically for important intellectual content. PE participated in the design of the study and helped drafting the manuscript. RvW supported data collection and participated in the design of the study. ME revised the manuscript critically for important intellectual content. All authors read and approved the manuscript.

## Pre-publication history

The pre-publication history for this paper can be accessed here:

http://www.biomedcentral.com/1471-2458/13/746/prepub
